# Kirigami-inspired multiscale patterning of metallic structures via predefined nanotrench templates

**DOI:** 10.1038/s41378-019-0100-3

**Published:** 2019-12-02

**Authors:** Mengjie Zheng, Yiqin Chen, Zhi Liu, Yuan Liu, Yasi Wang, Peng Liu, Qing Liu, Kaixi Bi, Zhiwen Shu, Yihui Zhang, Huigao Duan

**Affiliations:** 1 School of Physics and Electronics, State Key laboratory of Advanced Design and Manufacturing for Vehicle Body, Hunan University, 410082 Changsha, People’s Republic of China; 2 College of Mechanical and Vehicle Engineering, Hunan University, 410082 Changsha, People’s Republic of China; 3 AML, Department of Engineering Mechanics; Center for Flexible Electronics Technology, Tsinghua University, 100084 Beijing, People’s Republic of China

**Keywords:** Nanophotonics and plasmonics, Electrical and electronic engineering, Organic-inorganic nanostructures

## Abstract

Reliable fabrication of multiscale metallic patterns with precise geometry and size at both the nanoscale and macroscale is of importance for various applications in electronic and optical devices. The existing fabrication processes, which usually involve film deposition in combination with electron-beam patterning, are either time-consuming or offer limited precision. Inspired by the kirigami, an ancient handicraft art of paper cutting, this work demonstrates an electron-beam patterning process for multiscale metallic structures with significantly enhanced efficiency and precision. Similar to the kirigami, in which the final pattern is defined by cutting its contour in a paper and then removing the unwanted parts, we define the target multiscale structures by first creating nanotrench contours in a metallic film via an electron-beam-based process and then selectively peeling the separated film outside the contours. Compared with the conventional approach, which requires the exposure of the whole pattern, much less exposure area is needed for nanotrench contours, thus enabling reduced exposure time and enhanced geometric precision due to the mitigated proximity effect. A theoretical model based on interface mechanics allows a clear understanding of the nanotrench-assisted selective debonding behaviour in the peeling process. By using this fabrication process, multiscale metallic structures with sub-10-nm up to submillimetre features can be reliably achieved, having potential applications for anti-counterfeiting and gap-plasmon-enhanced spectroscopy.

## Ancient art informs cutting-edge manufacturing method

A centuries-old papercrafting technique has inspired an approach for the reproducible manufacture of metal structures with precisely defined macro- and nanoscale features. Many electronic devices incorporate such elements, but current production processes struggle with the ability to accurately sculpt both millimeter- and nanometer-sized design elements. Researchers led by Yihui Zhang of Tsinghua University and Huigao Duan of Hunan University tackled this challenge with a 21^st^ century version of ‘kirigami’, an ancient aesthetic pursuit of artistic paper-cutting. The authors used an electron beam to precisely cut multiscale patterns into a thin metallic film, and then used adhesive tape to peel away unwanted elements of the film. Using this technique, the authors demonstrate the capability to produce a range of designs with both sub-millimeter and nanometer-scale features, which could prove useful in applications including anti-counterfeiting or optical detection.

## Graphical abstract

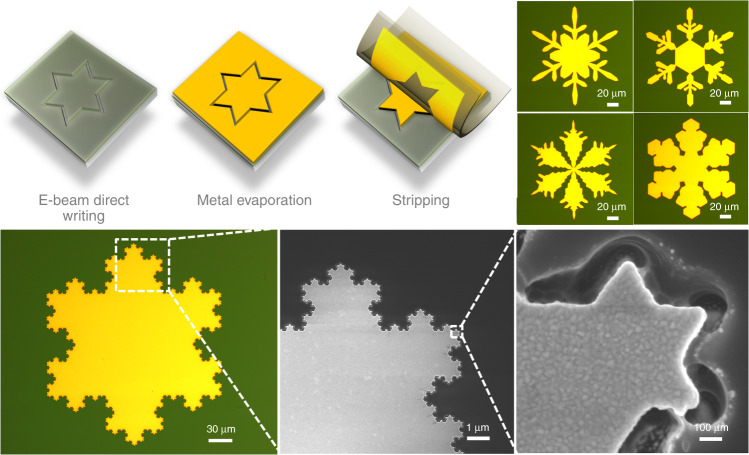

## Introduction

Multiscale structures including both macroscale and nanoscale features are essential for various applications in nanoelectronic and nano-optical devices^1–4^. In multiscale structures, the nanoscale features are usually used, based on the size effect, to improve the device performance or to interact more precisely with smaller objects such as molecules^5–7^, while the macroscale features are used to exchange electrical, optical or mechanical signals with the macroscopic world^8,9^. Considering the functionality, the most widely investigated multiscale structures are metallic structures with tiny gaps or sharp corners^10^. Among them, nanogap electrode pairs have attracted much attention for nanoelectronics and molecular electronics^11,12^, in which the gap size is supposed to be at the 10 nm or even 1 nm scale to achieve better device performance^13^, while the electrode pads are usually at the 100 μm scale for wire bonding^14^. Metallic nanogaps can also be used for focusing electromagnetic waves into the nanoscale to significantly enhance the light–matter interaction based on the plasmonic effects^15,16^. For example, researchers have demonstrated the funnelling of terahertz waves into sub-10-nm gaps with multiscale metallic structures, in which the microscale features serve as the resonators, while the nanogap provides the space to support the highly squeezed terahertz (THz) waves^17^. Similar to the multiscale metallic nanogaps, metallic structures with tiny corners are capable of modulating the electrical or optical field via the lightning rod effect^18,19^. A number of hierarchical metallic structures with fractal features^20^ and sharp tips have been proposed to maximize the field enhancement in plasmonic and electrostatic devices^21^. Theoretical calculations indicate that the electric field enhancement factor could be as high as 3 × 10^3^ when the radius of curvature is decreased to 5 nm^22^. The highly confined field is also useful for manipulating the field–matter interaction in an ultrasmall deterministic space, which is important for single-molecular analysis^23^.

Despite the broad interest, the reliable fabrication of multiscale structures with precise geometry and size at both the nanoscale and macroscale is challenging^24,25^. In most applications, 10-nm-scale features with 1-nm-scale precision are required to obtain satisfactory performance^26^. These high-resolution and precision requirements inevitably involve focused electron-beam processes, namely, electron-beam lithography (EBL)^27,28^. While EBL processes have shown the flexibility and capability to fabricate structures down to 5 nm, they are intrinsically not ideal tools for fabricating multiscale structures^29^. Because of its high-resolution point-by-point scanning process, the fabrication of multiscale structures with submillimetre features is highly time-consuming^30^. In addition, the proximity effect in EBL^31^ is drastically amplified in large patterns and thus severely degrades the precision of the nanoscale features.

To address the dilemma of EBL in terms of the precision and efficiency, we previously proposed a “sketch and peel” lithography (SPL) process, in which macroscopic structures can be realized by exposing only their contours using an electron beam^32^. Specifically, the contour is first defined using a high-resolution negative-tone resist hydrogen silsesquioxane (HSQ), which can split the subsequently deposited metallic film into different parts. The final structures can be obtained by selectively peeling the unwanted metals with the help of the HSQ nanowalls. Because much less exposure is required for the nanowall contours, the patterning efficiency can be improved, and the proximity effect can be mitigated. With this advantage, the SPL process has shown potential in fabricating various multiscale metallic structures and can also be extended to focused-ion-beam machining^33^. However, the current SPL process still has a couple of limitations in practical applications. For example, it is difficult to obtain ultrasmall metallic nanogaps due to the deformation of high-aspect-ratio HSQ nanowalls and the dewetting behaviour of metallic films. In addition, HSQ nanowalls have to be removed for many applications, but the removal of HSQ requires a hydrogen fluoride solution, which is harmful and incompatible with the commonly used SiO_2_ substrates^34^. More importantly, the yield of the process obviously decreases for structures larger than 25 μm, severely limiting its application for multiscale structures with submillimetre features. This limit is supposed to be intrinsically relevant to its debonding mechanics and dynamics and thus difficult to overcome.

Inspired by kirigami^35–38^, an ancient paper-cutting process, we propose and demonstrate a different electron-beam-based “SPL” process for multiscale metallic fabrication using predefined nanotrenches as the templates. Similar to the kirigami process, in which the final pattern is defined by cutting its contour in a paper and then removing the unwanted parts, we define the target multiscale structures by first creating nanotrench contours in a metallic film via an electron-beam-based process and then selectively peeling the isolated film outside the contours. Various kinds of multiscale metallic structures, such as fractals and nanogaps, have been demonstrated. The anti-peeling mechanism of the target structures was well revealed by finite-element-method-based modelling using interfacial fracture mechanics. Compared to the nanowall-based “SPL” process, this kirigami-inspired process demonstrates several advantages, such as higher yield for large structures with submillimetre features and the capability to obtain cleaner and smaller metallic nanogaps for optical-detection applications. Taking advantage of the selective peeling property of this process, exfoliable tags^39^ that include successive levels of security incorporating both covert and overt features were also demonstrated to have specific applications in the anti-counterfeiting field^40^. This work extends the capability of EBL and provides a new platform for creating multiscale metallic structures with precise geometry and size, which is supposed to have broad application potential in nanoelectronics and nano-optics.

## Results and discussion

**Fig. 1:**
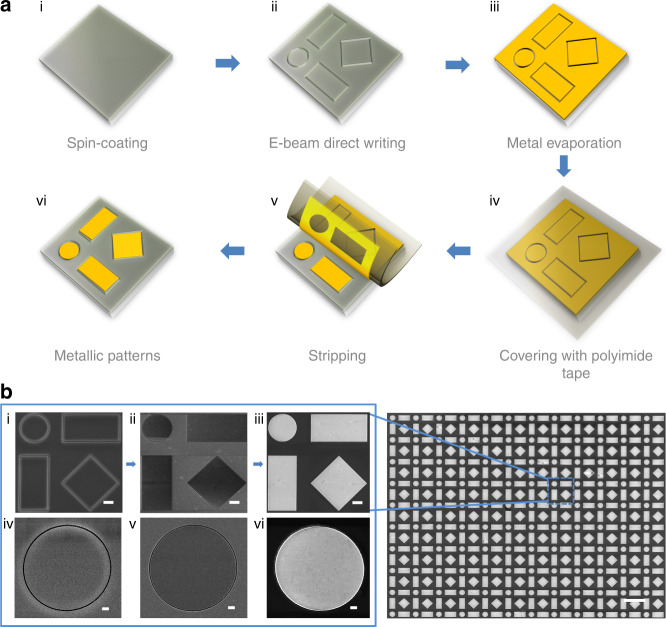
Fabrication flowchart of the kirigami-inspired process for metallic structures and its experimental demonstration. **a** Schematic of kirigami-inspired fabrication process flow. (i) PMMA resist spin-coated onto a silica substrate; (ii) single-pixel outline exposed by EBL process; (iii) direct metal deposition by electron-beam evaporator; (iv) deposited metallic film covered with PI tape; (v) detachment during the peeling process; and (vi) desirable metallic patterns attained by stripping the tape away. **b** Scanning electron micrographs of the periodic array. The unit cell of fabricated patterns to the left involves a circle, rectangles and a diamond. Enlarged images of a single unit cell before (i) and after (ii) metal film evaporation, respectively, and the resultant structures (iii) after stripping. Corresponding details for an individual circle shape are revealed from (iv) to (vi). Scale bar: 2 μm (**b** (i)–(iii)); 500 nm (**b** (iv)–(vi)); 20 μm (right inset in **b**)

Figure 1a intuitively illustrates a schematic flow of the fabrication procedure based on the kirigami-inspired electron-beam process. A poly (methyl methacrylate) (PMMA) resist was first spin-coated onto a SiO_2_/Si substrate (i). Then, EBL was conducted to obtain looped single-pixel nanotrenches (ii), which enabled the absolute separation of the internal and external PMMA layer. Subsequently, a noble metal film, for example, gold or silver without an adhesion layer, was deposited onto the sample (iii). The PMMA resist must be sufficiently thick to avoid the connection of the metallic thin films on the PMMA and in the nanotrenches. Then, the external metallic film can be peeled off by a polyimide (PI) tape (iv and v) due to the weak adhesion of the deposited metal film on the underlying PMMA layer^41^ compared with that between the adhesive polymer and the metal thin film. However, the internal metallic film can well remain due to its sufficient stability, as discussed later in the simulation part. Therefore, by defining the outlines of nominal structures by looped curve exposure and subsequent selective peeling, expected metal structures were generated (vi). The unexposed PMMA layer remained on the substrate surface after the peeling-off process. Note that unlike kirigami art, which requires scissors to directly cut the paper sheets, the “cutting” process for the nanotrenches was applied to the polymer resist before depositing the metal film rather than directly acting on the metal film.

To illustrate the feasibility and versatility of this approach for micro/nanopatterning, an array consisting of gold patterns with three different kinds of shapes (rectangle, circle and diamond) was fabricated on a silica substrate; the corresponding scanning electron microscopy (SEM) images are shown in Fig. 1b (right). The successful implementation of large-area structures with various shapes was demonstrated. Meanwhile, the time-consumption issue, the well-known challenge for EBL-based patterning, was eliminated by single-pixel outline exposure. Enlarged SEM images of a unit corresponding to the process in Fig. 1a (ii), (iii) and (vi) are shown in Fig. 1b (i), (ii) and (iii), respectively. More details of the disk patterns can be found in Fig. 1b (iv), (v) and (vi). The obtained large-area structures with high fidelity indicate the feasibility of the proposed kirigami-inspired approach. In the process, the EBL system is able to create a smooth circle edge for forming internal patterns, which is significant for subsequent dry peeling. To intuitively demonstrate the feasibility of this peeling-off process on a macroscopic scale, the whole process for a periodic 1-mm-diameter gold disk is given in Fig. S1.

**Fig. 2:**
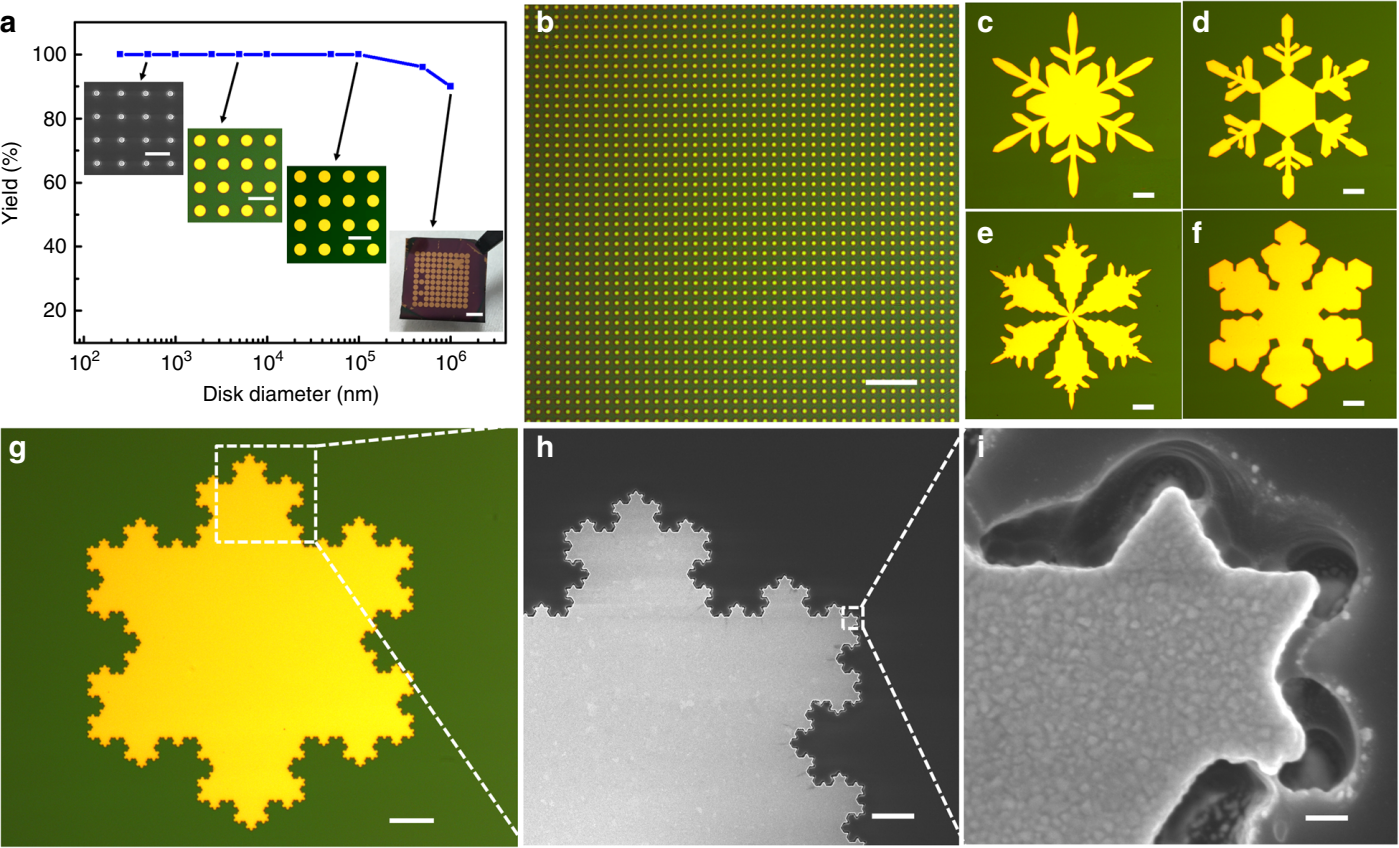
Yield and implementation of complicated architectures. **a** Plot of yield versus disk diameter, which ranges from 250 nm to 1 mm. Inset shows graphs of partial arrays. **b** The uniformity of the 1-μm-diameter disk pattern defined by the kirigami-inspired process. **c–f** Optical photographs of a series of symmetric snowflake-like patterns. **g** Optical image of self-similarity Koch snowflake fractal antenna. **h** An enlarged SEM micrograph of the Koch structure indicated by the white dashed square in **g**. **i** High-resolution SEM image of the selected region in **h**, showing a curve radius of ~20 nm. Scale bar: 2 μm, 10 μm, 100 μm, and 3 mm (inset in **a** from left to right); 5 μm (**b**); 20 μm (**c**–**f**); 30 μm (**g**); 1 μm (**h**); and 100 nm (**i**)

The kirigami-inspired process is capable of defining submillimetre structures reliably. Figure 2a presents the yield of this peeling process as a function of the diameter of the disks. A 100% yield was obtained for the array when the diameter of the disk varied from 500 nm to 100 μm, and even up to an 88% yield was obtained for a 1-mm-diameter periodic disk array (see detailed corresponding micrographs in Fig. S2). The inset shows four micrographs of the obtained arrays with four typical sizes. The periodic gold holes attached to the adhesive tape reveal the thorough removal of the external gold layer during stripping, as shown in Fig. S3. A bright-field photograph of the 1-μm-diameter gold disk array shown in Fig. 2b reveals the high uniformity of the structures. To further illustrate the capability of this process for complicated multiscale structures, a series of snowflake-like patterns was designed for fabrication. The results are shown in Fig. 2c–f, in which the structures have a lateral dimension of 150 μm. This confirms the compatibility of the kirigami-inspired process for the rapid fabrication of arbitrary metallic micro-nanopatterns with clear concave and cornered features. Koch fractal geometries (250 μm × 250 μm area) with complex self-similarity, which are candidates as antennas for compact communication devices^21^, were also fabricated to demonstrate the multiscale fabrication capability of the process, as shown in Fig. 2g. The key features of this fractal structure cross four orders of magnitude, with a lateral dimension of 250 μm, but the smallest feature is at the 10 nm scale. These structures with multiscale features across the 10 nm to submillimetre scale are extremely difficult to define with conventional point-by-point processes due to the proximity effect. With this process, sharp corners with a curvature radius of ~20 nm were obtained in the Koch snowflake fractal antenna, as shown by the details of selected areas in Fig. 2h, i, indicating the advantage of the process. Note that the distortion of the unexposed PMMA near the trench was caused by electron-irradiation-induced damage during the SEM imaging^42^.

**Fig. 3:**
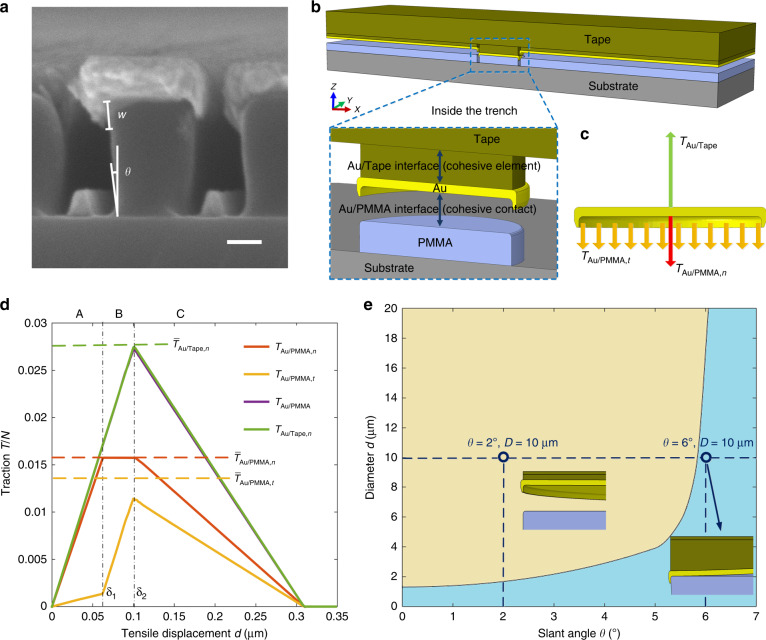
Underlying mechanism of the kirigami-inspired process. **a** SEM of cross-sectional view of PMMA-based grating after gold evaporation defined on SiO_2_ substrate. **b** Schematic of the FE model with no slant angle in this simulation. The Au outside the trench is pulled out, whereas the gold disk inside the trench is not. The inset shows the details of interfaces inside the trench, where the gold disk and PMMA are separated for a better view. **c** Diagram of force analysis for the Au disk in **b**. **d** Plot of calculated tractions versus tensile displacement during the entire process and the corresponding thresholds. **e** Phase diagram in terms of the diameter and slant angle, where the light blue region means the Au disk is left on the PMMA and the light brown region means the opposite. Here, the two insets denote the states under *θ* = 2° and *θ* = 6° with a fixed diameter, *D* = 10 μm. Scale bar: 100 nm (**a**)

Selective peeling of the external metal film is essential for this kirigami-inspired process. To understand this interesting behaviour, a quantitative mechanics modelling of the interface fracture was carried out based on finite-element analysis (FEA; see Methods section for details). The morphology of the structures based on the experimental observations was exploited to construct the geometric model in the simulations. Figure 3a provides a typical cross-sectional SEM of a PMMA-based grating after gold evaporation, in which the undercut structure (PMMA) shows a slant angle of *θ*, typically in the range of 0° and 8°. Due to the clogging effect^43,44^ during metal deposition, the side surfaces (with a height of *w*) of the PMMA in the vicinity of its top were also covered by gold. Figure 3b shows an illustration of an FE model with no slant angle containing a pattern of 1-μm-diameter roundness at the centre. The intrinsic mechanism of the peeling process is the competition between the fracture of the Au/Tape and Au/PMMA interfaces, which is described by the cohesive zone model shown in Supplementary Information 4(I). It should be noted that a pre-crack was introduced in the Au/PMMA interface outside the trench, which gradually propagated in the subsequent steps^45^, ensuring that the associated gold film surrounding the features was stripped down. Therefore, we focus on the pattern zone inside the trench, mainly involving a gold disk, the associated PMMA cylinder and the tape. Since the feature size (1–20 μm in the following analysis) was much less than the curvature radius (~200 μm) of the tape, the tape around the individual feature remained approximately horizontal during the entire crack propagation process. As such, the failure of the Au/Tape interface was mainly governed by the mode-I cracking in terms of the crack opening. In contrast, the failure of the Au/PMMA interface at the top and side surfaces of the PMMA (referred to as the top and side Au/PMMA interfaces for simplicity) were governed by the mode-I cracking and mode-II cracking (in terms of crack shearing), respectively. Although the Au/Tape interface was stronger than the top Au/PMMA interface, the competition between the Au/Tape interface and Au/PMMA interface was still unclear, as the strength of the mode-II cracking was usually much larger than that of the mode-I cracking, and the pull-out of the gold at the side PMMA surface needed to overcome the elastic deformation energies induced by the slant sidewall.

For a nearly vertical sidewall of the PMMA with relatively small dimensions (e.g. <1 µm), we can employ theoretical analyses to estimate a critical diameter for a successful kirigami-inspired process. Under this condition, the normal forces at the side PMMA surface essentially did not resist the pull-out process. The stress states of all interfaces can be assumed to be uniformly distributed, considering the small disk diameters. All forces acting on the Au disk are demonstrated in Fig. 3c, including two normal tractions, $T_{{\mathrm{Au}}/{\mathrm{Tape}},n}$ and $T_{{\mathrm{Au}}/{\mathrm{PMMA}},n}$, and a tangential traction $T_{{\mathrm{Au}}/{\mathrm{PMMA}},t}$, where $T_{{\mathrm{Au}}/{\mathrm{PMMA}},n}$ and $T_{{\mathrm{Au}}/{\mathrm{PMMA}},t}$ were applied at the top and side Au/PMMA interfaces, respectively. Traction–tensile displacement curves based on simulation results of the model in Fig. 3b are shown as the solid lines in Fig. 3d, whereas the dashed lines correspond to failure thresholds. Detailed analyses of these results (see Supplementary Information 4(II)) suggest that the gold disk was not pulled out in this case, mainly because of the resistance ($T_{{\mathrm{Au}}/{\mathrm{PPMMA}},t}$), although $T_{{\mathrm{Au}}/{\mathrm{PMMA}},n}$ had reached its threshold. This is described by the following condition:

1 $$\bar T_{{\mathrm{Au}}/{\mathrm{PMMA}},n} + \bar T_{{\mathrm{Au}}/{\mathrm{PMMA}},t} \ge \bar T_{{\mathrm{Au}}/{\mathrm{Tape}},n},$$

where the two normal traction thresholds ($\propto\!\! D^2$) grew faster than the tangential threshold ($\propto \!\!D$) according to the scaling law. Combining Eq. (1) and Eq. (S1) (see Supplementary Information 4(II) for details), we obtain

2 $$D \,{\le \frac{{4\bar \sigma _{{\mathrm{Au}}/{\mathrm{PMMA}},t}}}{{\bar \sigma _{{\mathrm{Au}}/{\mathrm{Tape}},n} - \bar \sigma _{{\mathrm{Au}}/{\mathrm{PMMA}},n}}}w}.$$

Equation (2) shows that to ensure a successful kirigami-inspired process, the disk diameter *D* should be below a critical value of ~1.2 μm for the material system considered herein.

When the slant angle was non-negligible and the feature size was relatively large, the critical diameter was substantially increased, mainly due to the contributions of the normal forces at the side PMMA surface. Additionally, the failure in the middle of the top Au/PMMA interface could appear prior to the edge location. Analyses of the detailed failure process can be found in Supplementary Information 4(III), where we concluded that the threshold of the diameter was mainly determined by the resistance induced by the slant sidewall. Since the slant angle was too small to be measured precisely by the SEM image, a series of simulations with *θ* ranging from 0° to 8° was performed. These results are demonstrated in a phase diagram (Fig. 3e), where the light brown region means the gold disk was pulled out and the light blue region means the opposite case. It is noteworthy that the allowable resistance force increased sharply beyond *θ* ≈ 5.5°. This arose mainly from the slant sidewall that possessed a strong constraint, especially at relatively large slant angles, such that the pull-out process needed to overcome much more additional strain energy (see Supplementary Information 4(III)–(IV) and Fig. S5 for details). For a slant angle of *θ* = 7°, the critical diameter can be estimated to be a couple hundred micrometres, at the same order of magnitude as that of the experimental observations.

**Fig. 4:**
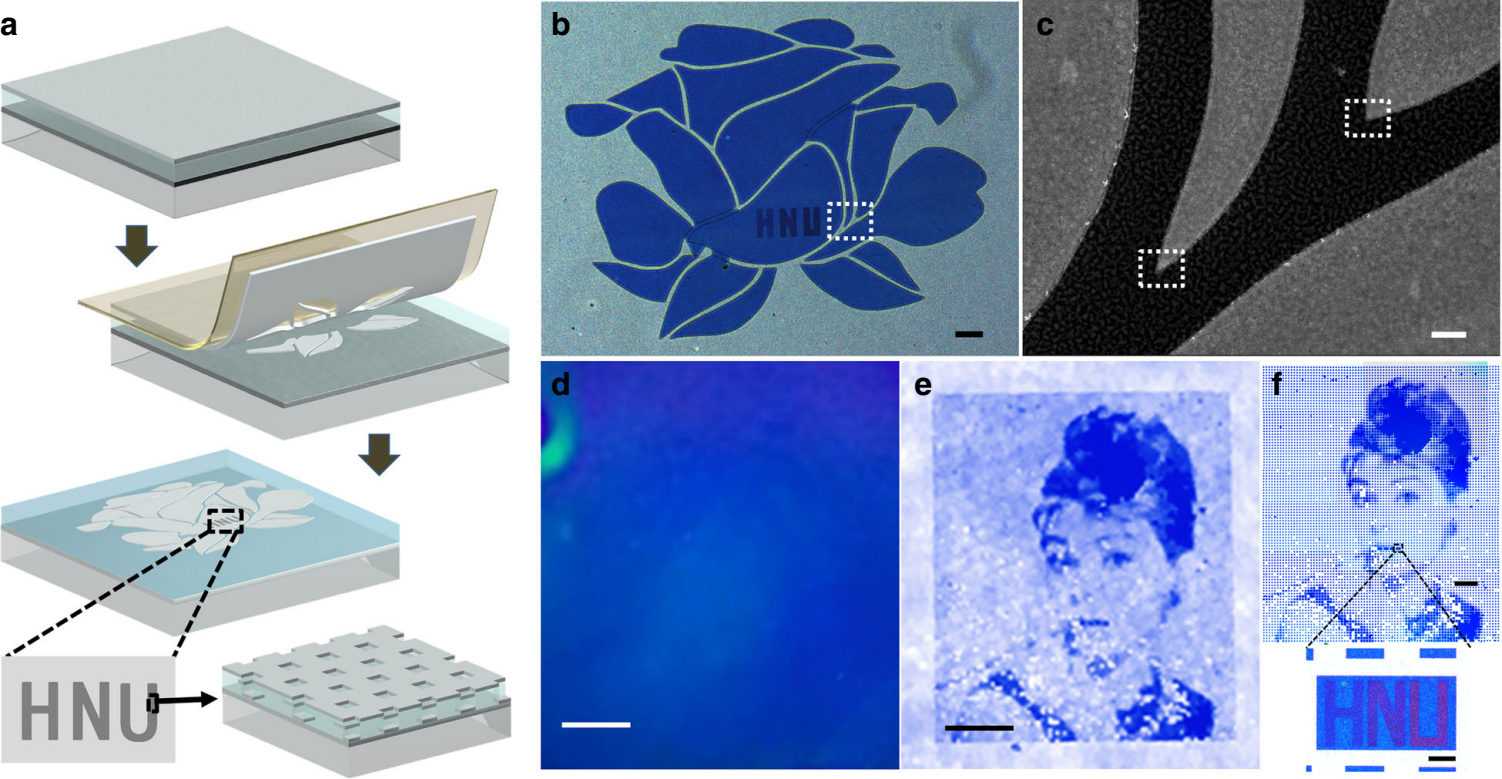
Potential applications as exfoliable labels for steganography. **a** Schematic flow of exfoliable labels for anti-counterfeiting applications. Inset shows the detailed information of concealed plasmonic silver nanoholes within an individual micro-pixel pattern. **b** Optical image of a rose flower after peeling off the top unexpected silver film. **c** The corresponding enlarged electron micrograph of the labelled area. **d**, **e** Digital photographs showing a visible greyscale pattern of a portrait of Audrey Hepburn before (**d**) and after (**e**) stripping. **f** Transmission optical micrograph of the vivid miniature. Inset shows the colourful miniature consisting of the “HNU” characters concealed in the lip. Scale bar: 30 μm (**b**); 1 μm (**c**); 1 mm (**d**, **e**); 400 μm (**f**); and 20 μm (inset in **f**)

We then demonstrate the potential applications of this multiscale patterning process. As a possible specific application, the selective peeling behaviour of this process provides a rapid fabrication strategy for obtaining exfoliable labels for steganography. The basic idea is shown in Fig. 4a; before peeling, the label looks like a homogeneous metallic film because the contours of the latent patterns are almost invisible due to the 10 nm scale dimension. After selective peeling, the latent patterns become visible. More interestingly, when defining the contours using the electron beam, nanostructures such as nanoholes can be simultaneously defined in the patterns, providing extra finer features for information encryption^46,47^, as shown in the inset. Note that the tape for peeling can be integrated with the sample in practical applications. As a demonstration, Fig. 4b shows a label containing a latent rose obtained after selective peeling, in which the vivid flower has a lateral dimension of 200 μm. The initial label, which includes latent patterns inside, is obtained using the kirigami-inspired process. The fabrication of this rose pattern costs only ~1 min to expose the outlines of the petals, which is 10 times faster than the conventional point-by-point exposure process. Note that the exhibited sapphire blue colour of the sample was generated by a metal–dielectric–metal Fabry–Perot (MDMFP) cavity^48^ consisting of a 220-nm-thick PMMA layer embedded in two sets of 30-nm-thick silver thin films. In this process, by taking advantage of the transparent dielectric property of the PMMA resist, the MDMFP cavity was intentionally introduced to demonstrate the feasibility of this process for colourful exfoliable labels. The corresponding enlarged SEM image denoted by the square dashed line in the rose is shown in Fig. 4c. Highlights reveal the high fidelity of the sharp corner in large patterning using the kirigami-inspired process. Three characters, “HNU”, the abbreviation of Hunan University, were embedded in a piece of the flower. The characters have a microscale size and are composed of subwavelength plasmonic elements, indicating the advantage of this process for multiscale fabrication. This kind of high-security tag includes successive levels of security incorporating both covert and overt features that are supposed to have applications in anti-counterfeiting. For instance, it can possess dual security levels, one readable by the naked eye and another readable only by dedicated digital readers. Enabled by the significantly enhanced fabrication efficiency, this process has the capability to directly obtain centimetre-scale greyscale pictures for naked eye observation. As a demonstration, we investigated a large-area exfoliable label exhibiting a portrait of Audrey Hepburn based on a fabrication architecture similar to that of the rose mentioned above. Figure 4d shows a transmission photograph of the actual label taken by a digital camera before peeling the adhesive tape. An artificial light source is illuminated from the backside of the label. There is no readable information but a uniform bright sapphire blue colour induced by the FP cavities. After exfoliation, a greyscale picture with an area of 4 mm × 6 mm emerges, as shown in Fig. 4e, consisting of microscale silver squares with various filling factors, corresponding to the RGB values obtained from the original picture. The micro-squares were fabricated by predefined contours using transparent PMMA. Notably, the large-area greyscale picture is readable by the naked eye. An optical microscope was used to obtain a detailed and distinct micrograph. As shown in Fig. 4f, a more vivid portrait that is blue in colour was observed. It should be noted that the difference in the light source and imaging tool led to the slight chromatic difference between Fig. 4e, f. Note that some portions of the image were missing, possibly owing to the pattern defects during the large-area exposure on the insulating glass substrate. In the inset, the colourful miniaturized “HNU” concealed in the lip can be observed only by a magnifying optical microscope. The initial colour image display was constructed using silver plasmonic arrays. More information about the optical properties and characterization of the tags is shown in Fig. S6. We believe that this concept for security tags can be mass-produced using nanotrench templates fabricated by soft printing or nanoimprint lithography techniques.

**Fig. 5:**
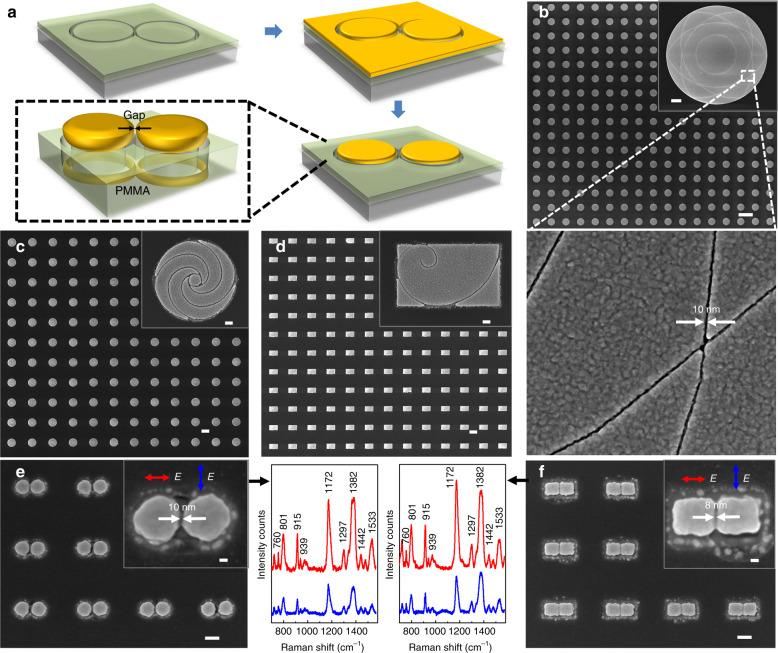
Metallic patterns with tiny nanogaps for optical and sensing applications. **a** Fabrication flow and the 3D schematic of the final metallic nanogap. **b** A metallic plate array with a diameter of 10 μm containing sub-10-nm gaps for potential THz device applications. The enlarged SEM image shows the uniform gaps inside. **c**, **d** One-micrometer metallic elements with a sub-10-nm gap based on Fibonacci spiral. **e**, **f** Plasmonic dimers with a size of 100 nm and a sub-10-nm gap, with arrows pointing to the Raman measurements under the illumination of horizontal (red) and vertical (blue) laser excitation. The pitch between dimers is 400 nm. Scale bar: 20 μm (**b**); 1 μm (inset in **b**); 1 μm (**c**, **d**); 100 nm (inset in **c**, **d**); 100 nm (**e**, **f**); and 20 nm (inset in **e**, **f**)

This kirigami-inspired process also has the advantage of defining metallic nanogaps in multiscale structures. Metallic nanogaps are the basic building blocks in nanoelectronics and nanoplasmonics^5,49^, but their reliable fabrication with the conventional electron-beam-based lift-off or etching process is very challenging because of the electron-scattering-induced proximity effect when exposing the multiscale structures. The kirigami-inspired process, on the one hand, can obtain narrower trenches on the positive-resist template due to the significantly mitigated proximity effect. On the other hand, no additional lift-off or etching process is required. More interestingly, the metallic nanogaps tend to decrease during the metal deposition process because of the aforementioned clogging effect. Notably, provided by the proper thickness of the deposited metal film, the gap can avoid inter-connection, which can deteriorate the device performance. As an example, we demonstrate the capability of the kirigami-inspired process for the efficient and reliable definition of sub-10-nm metallic nanogaps for near-field optics and plasmonic applications. Figure 5a shows the fabrication process flow for the metallic nanogaps and the 3D schematic illustration of the final nanogaps. Starting from nanotrenches with shared boundaries, the metallic patterns are defined by the internal isolated PMMA templates after selective peeling, and the nanogaps form at the shared boundaries. Various metallic nanogaps were demonstrated by this strategy. Figure 5b exhibits an array consisting of 10-μm-diameter plates with flower-like trench textures. Uniform nanogaps with a size of ~10 nm are distributed along the surface of the gold thin film, as indicated in the enlarged SEM image highlighted by the dashed white line. This large-area multiscale architecture containing extremely tiny gaps provides a promising platform for optical detection in the THz regime^17^. Figure 5c, d present arrays of metallic patterns based on Fibonacci spiral elements with sub-10-nm gaps, implying potential in the fabrication of specific chiral structures for nano-optical devices. Notably, residual gold particles around the PMMA nanotrenches shown in the inset can be observed, which might be caused by incomplete removal of the metallic thin film during the tape stripping process.

To demonstrate the role of tiny nanogaps for applications, plasmonic nanodisk and nanosquare dimers with sub-10-nm gaps were defined for surface-enhanced Raman spectroscopy (SERS)^50^ using the kirigami-inspired process, as shown in Fig. 5e, f, respectively. Apparent polarization-dependent SERS spectra of crystal violet molecules are shown in the insets. The enhanced Raman signals occur when structures were illuminated by a laser parallel with the gap, indicating the strong plasmon coupling supported by the tiny nanogaps. The measured and calculated scattering spectra and near-field distribution are shown in Fig. S7, further confirming the key role of the nanogaps. This fabrication strategy is also applicable to transparent substrates for optical applications in the infrared region, as shown in Fig. S8. The polarization-dependent infrared absorption spectra were also obtained in the nanogapped structures due to plasmon coupling, which is supposed to have potential applications in surface-enhanced infrared spectroscopy.

## Conclusions

In conclusion, we proposed a kirigami-inspired electron-beam patterning approach for defining multiscale metallic structures. The approach is realized by selectively stripping an unwanted metallic film using predefined nanotrenches as the templates without involving any pattern transfer and resist removal steps. Compared with the conventional electron-beam process, this kirigami-inspired approach has the advantages of significantly enhanced patterning efficiency and geometric precision because only single-pixel contours of the final patterns are exposed. However, note that the existence of an underlying PMMA resist is incompatible with subsequent cleaning processes for some specific applications; hence, additional metallic pattern transfer is necessary. Meanwhile, because small individual soft PMMA nanoposts separated from the surrounding metal film by nanotrench contours can fall over towards adjacent structures, metallic features with dimensions of less than tens of nanometres are more difficult to obtain by using this strategy. Nevertheless, systematic experiments confirmed the feasibility and reliability of this approach for multiscale structures. A numerical model based on the interfacial fracture mechanics was used to understand the selective adhesion-debonding behaviour, and the simulation results well explain the experimental results. By utilizing the advantages of selective peeling, high-resolution sub-10-nm metallic gaps with enhanced fabrication throughput and several types of multiscale metallic structures were demonstrated for anti-counterfeiting and high-sensitivity optical-detection applications. We believe that the fabrication strategy can be further extended to other metals for broader applications via adhesion engineering.

## Methods

### Layouts and lithographic dose set

The nominal patterns were designed to consist of single-pixel closed-loop outlines. Lithographic doses in layouts are different for patterns in this work according to the dimensions of the shapes. In Fig. 1, the exposure dose was set to 2.5 nC/cm. In the yield graph in Fig. 2a, the dose parameters are 1.8 nC/cm for 250 and 500 nm, 2 nC/cm for 1 μm, 2.2 nC/cm for 5 μm, 2.5 nC/cm for 10 μm, 4 nC/cm for 50 and 100 μm, 7 nC/cm for 500 μm and 8 nC/cm for 1 mm. In Fig. 2c–g, the same dose of 2.2 nC/cm was used for both the Koch fractal geometries and snowflake structures. Moreover, the dose used to define the outline of the latent rose and portrait was set to 5 nC/cm and 500 μc/cm^2^, respectively, for the plasmonic nanoholes inside. In Fig. 5, to achieve structures with a high-resolution ultra-tiny gap, a thinner resist and a low temperature development were used instead, and the dose was increased. The dose was 2.5 nC/cm for various structures and 1 nC/cm for the shared defining outline to achieve the tiniest nanogap in the dimer configuration.

### Electron-beam lithography

Patterning was conducted on a 10 mm × 10 mm square silicon oxide substrate (with 285 nm of SiO_2_ on a silicon substrate). PMMA with a molecular weight of 950k was deposited by spin-coating a solution of 6% 950k PMMA in anisole at 4k r.p.m. and pre-baking in a hot plate at 180 °C for 5 min. The thickness of the PMMA layer was determined to be 434 nm by an ellipsometer. Single-pixel outline exposure was conducted by a Raith 150^two^ EBL system at a 30 kV acceleration voltage, with a 60 μm aperture and using a beam current of 1150 pA. The PMMA was developed at room temperature (20 °C) for 1 min in a 3:1 solution of isopropyl-alcohol:methyl isobutyl-ketone, immersed in IPA for 1 min, and then dried under a N_2_ stream. To increase the resolution of the single-pixel line, a 30 μm aperture and a beam current of 260 pA were used for exposure, and the exposed thinner resist (3% 950k PMMA, 2k r.p.m., 80 nm thickness) was developed at −17 °C for structures with tiny gaps in Fig. 4.

### Metal deposition

The metallization of nanotrench-based patterns was conducted by an electron-beam evaporator (Kurt J. Lesker, Lab-line). To obtain smooth and continuous thin films, the vacuum chamber was pre-pumped to 10^−7^ Torr, and the actual press during the gold evaporation process was maintained at 10^–6^ Torr with a deposition rate of 0.5 Å/s. The thicknesses of the gold and silver thin films were set to 40 and 30 nm, respectively, as monitored by a quartz-crystal microbalance.

### Morphological characterization

The micrographs of the metallic patterns were obtained by a field-emission SEM (Carl-Zeiss sigma HD) at an acceleration voltage of 10 kV and a working distance of 6 mm. The optical characterization was acquired by a Carl-Zeiss microscope (AXIO-10) equipped with ×5 (0.13 numerical aperture (NA)), ×10 (0.25 NA), ×20 (0.4 NA), ×50 (0.75 NA), and ×100 (0.85 NA) objective lens.

### Mechanics simulations

An FEA model was built to simulate the peeling processes using the conventional static analysis in the commercial software ABAQUS (v6.14, Simulia Inc., Providence, RI). Without loss of generality, the mechanical model of a periodic unit was analysed based on a global Cartesian coordinate system, with the *z*-axis aligned with the direction of the thickness and the *x*-axis aligned with the direction normal to the macroscopic crack front. Owing to the symmetry with respect to the *xz*-plane, only a half part was simulated in the FEA. The elastic modulus (*E*) and Poisson’s ratio (*ν*) were $E_{\mathrm{PMMA}} = 2\,{\mathrm{GPa}}$ and $\nu _{\mathrm{PMMA}} = 0.34$ for PMMA; $E_{\mathrm{Au}} = 77.2\,{\mathrm{GPa}}$ and $\nu _{\mathrm{Au}} = 0.42$ for Au; and $E_{\mathrm{PI}} = 2\,{\mathrm{GPa}}$ and $\nu _{\mathrm{PI}} = 0.34$ for the tape. All the interface parameters (e.g. interface strength and interface energy) were tested by the peeling test and tensile test, including $W_{{\mathrm{ad}},{\mathrm{Au}}/{\mathrm{tape}}} = 54\,{\mathrm{N/m}}$,$W_{{\mathrm{ad}},{\mathrm{Au}}/{\mathrm{PMMA}}} = 24\,{\mathrm{N/m}}$, $\bar \sigma _{{\mathrm{Au}}/{\mathrm{tape}},n} = 0.07\,{\mathrm{MPa}}$, $\bar \sigma _{{\mathrm{Au}}/{\mathrm{PMMA}},n} = 0.04\,{\mathrm{MPa}}$ and $\bar \sigma _{{\mathrm{Au}}/{\mathrm{PMMA}},t} = 0.24\,{\mathrm{MPa}}$. The PMMA and tape were modelled as incompressible neo-Hookean solids (C3D8RH, C3D6H, C3D4H in ABAQUS), and the Au, as a continuum shell (SC8R, SC6R in ABAQUS). The cohesive behaviour was employed through the contacting interface property for the Au/PMMA interface and through a layer of cohesive element (COH3D8 in ABAQUS) for the Au/Tape interface. The accuracy of the computation was ensured by refined meshes. The bottom surface of the PMMA was fixed, and to the top surface of the tape was applied a prescribed displacement loading along the +*z* direction.

### FDTD simulations

Three-dimensional finite-difference time-domain simulations (Lumerical Solutions Inc., Version 8.15) were performed to calculate the optical responses and near-field distribution profiles. The refractive index of the quartz substrate and PMMA were set to 1.48 and 1.46, respectively. The dispersion model of gold was based on the data from Johnson–Christy. Perfectly matched layer (PML) boundary conditions were used for the three dimensions to simulate the scattering intensity of the dimers. The mesh sizes used were 1, 1 and 3 nm along the *x*, *y* and *z* coordinates, respectively. In contrast, periodic boundary conditions along the *x*-axis and *y*-axis and the PML along the propagation of the electromagnetic waves (*z*-axis) were used to calculate the transmission spectra of nanodisk dimers on the quartz substrate.

### Raman measurement

The prepared samples of the 100-nm-diameter gold nanodisk and nanosquare dimer array defined on the SiO_2_/Si substrate were immersed in a solution of crystal violet diffused in ethanol at a concentration of 10^−5^ M for 18 h and finally blow-dried by a steady N_2_ stream to obtain an average single-molecule layer. The Raman signals were collected by a microspectrometer (Andor) through a ×100 (0.85 NA) microscope objective (Leica) equipped with a 632.8 nm laser (16 μW, ~1-μm-diameter spot size). The measurements were obtained with an integration time of 5 s ten times.

### Near-field optical characterization

An optical microscope (Carl-Zeiss Axio-10) equipped with ×5 (0.13 NA), ×10 (0.25 NA), ×20 (0.4 NA), ×50 (0.75 NA), and ×100 (0.85 NA) objective lens is used to obtain the reflection and transmission optical micrographs with different magnifications. The transmittance spectra under normal incidence were collected by an Olympus microscope (BX-51) with a spectrometer through a ×100 objective (MPlan-FLN, 0.9 NA). The extinction spectra were measured in transmission mode using a 2030 PV ultraviolet–visible–near-infrared range microspectrophotometer (CRAIC Technology Inc.) equipped with a xenon light source (80 W) and an optical objective (ZEISS Ultrafluar, ×10, 0.2 NA). Both the incident and collected light were normal to the quartz substrate, thus providing linearly polarized light excitation in plane with the surface of the nanostructures.

## Supplementary information

Supplementary Information-clear
