# Supplementary material for: Kirigami-inspired multiscale patterning of metallic structures via predefined nanotrench templates
Source: Microsyst Nanoeng. 2019 Dec 2;5:54. doi: 10.1038/s41378-019-0100-3 (PMC6885514; doi:10.1038/s41378-019-0100-3)
Supplement: Supplementary file 1 — Supplementary Information-clear [file 41378_2019_100_MOESM1_ESM.docx]

***Supplementary Information of***

**Kirigami-Inspired Multiscale Patterning of Metallic Structures *via* Predefined Nanotrench Templates**

Mengjie Zheng^1, 2, #^, Yiqin Chen^2, #^, Zhi Liu^3, #^, Yuan Liu^3^, Yasi Wang^2^, Peng Liu^2^, Qing Liu^2^, Kaixi Bi^1^, Zhiwen Shu^2^, Yihui Zhang^3, *^, Huigao Duan^1, *^

^1^ School of Physics and Electronics, State Key laboratory of Advanced Design and Manufacturing for Vehicle Body, Hunan University, Changsha 410082, P. R. China.

^2^ College of Mechanical and Vehicle Engineering, Hunan University, Changsha 410082, P. R. China.

^3^ AML, Department of Engineering Mechanics; Center for Flexible Electronics Technology, Tsinghua University, Beijing 100084, P. R. China.

# These authors contributed equally to this work.

* Correspondence: Huigao Duan ([duanhg@hnu.edu.cn](mailto:duanhg@hnu.edu.cn)) and Yihui Zhang ([yihuizhang@tsinghua.edu.cn](mailto:yihuizhang@tsinghua.edu.cn))

1. **Macroscopic behavior of 10×10 1-mm-diameter gold disk array during overall SPL process**.


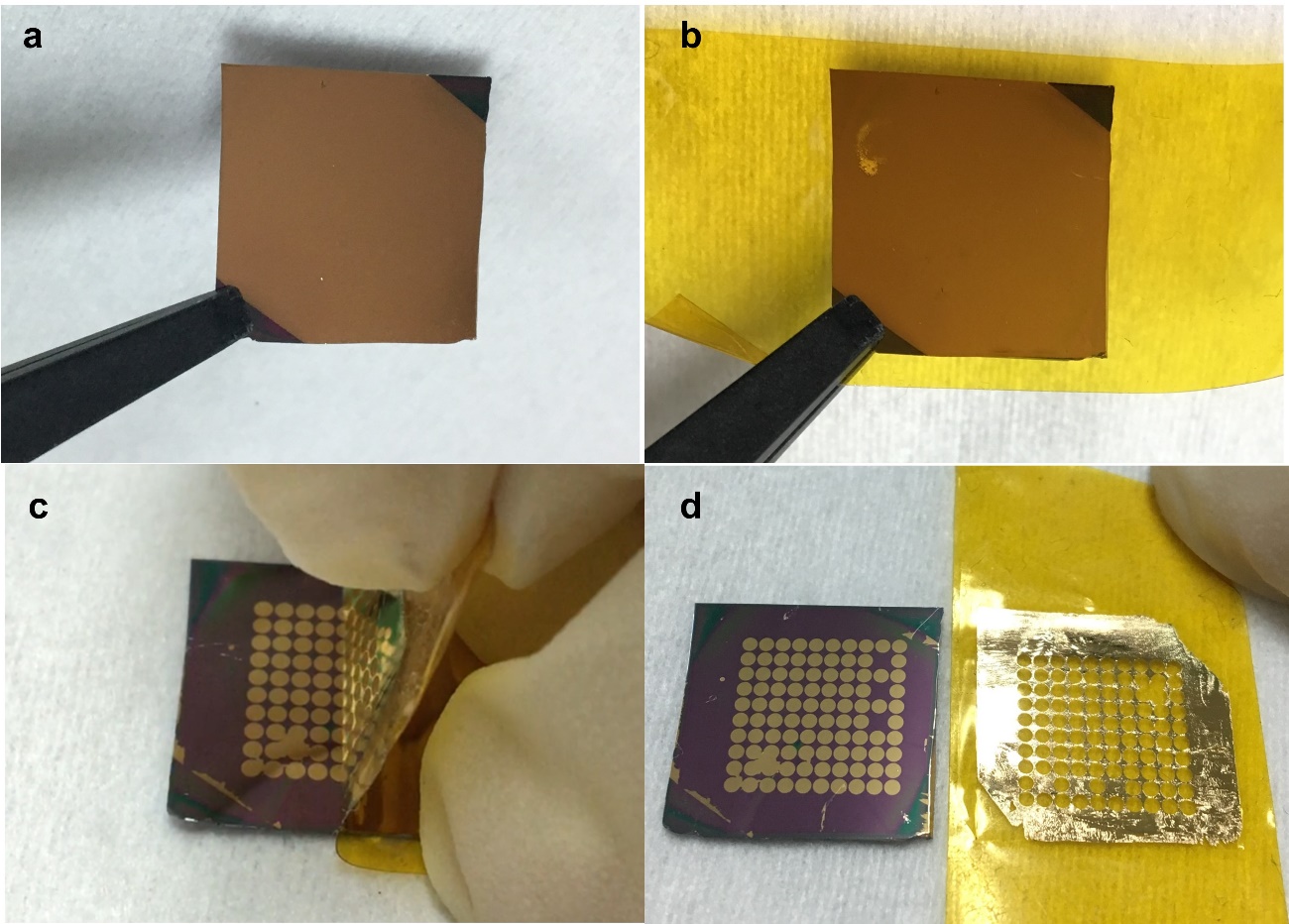


**Fig. S1 a**, Specimen after gold evaporation. **b**, Covering with PI tape on the surface of gold film. **c**, Stripping off process. **d**, Comparison of gold disks on SiO_2_ substrate and gold holes attached on the adhesive tape.

1. **The SEM images and photographs of gold disk array with different diameter ranging from 500 nm to 1mm.**

**
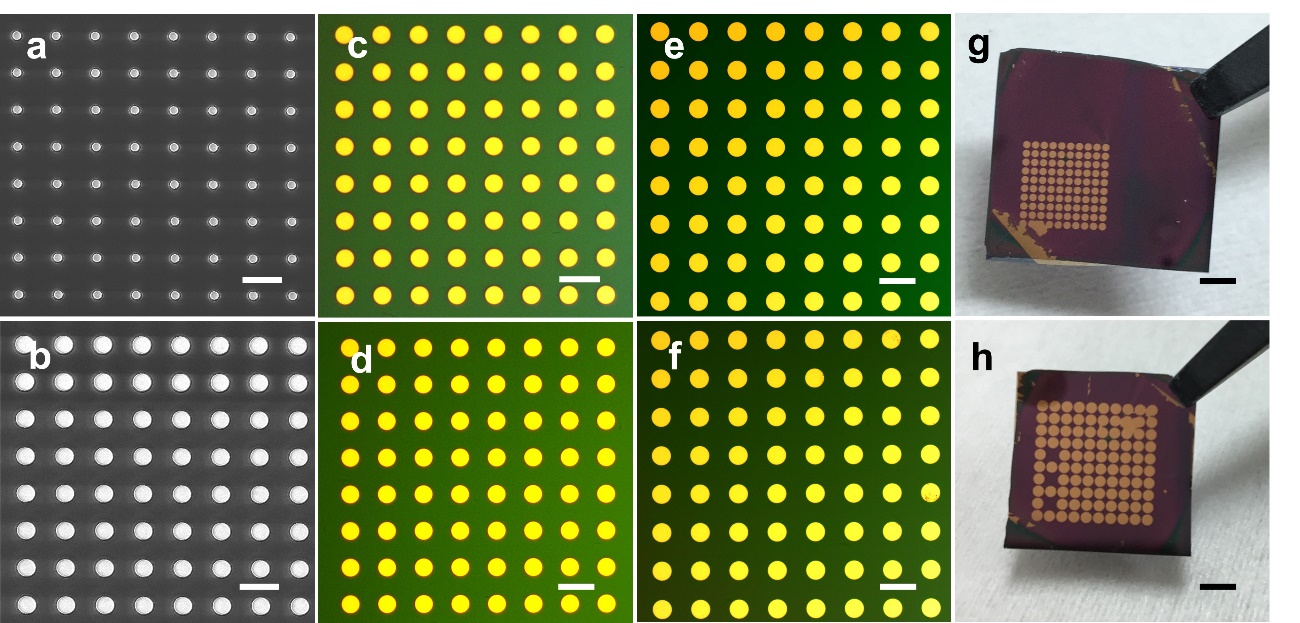
**

**Fig. S2 a-b,** Scanning electron characteristics of disk array with 500 nm and 1 μm diameter. **c-f,** Optical images of 5 μm, 10 μm, 50 μm and 100 μm diameter array. **g-h,** Digital images of 500-μm- and 1-mm-diameter disk array. Scale bar: 2 μm (a and b), 10 μm (c), 20 μm (d), 100 μm (e), 200 μm (f), 2 mm (g), 3 mm (h).

1. **Optical images of periodic gold holes with different diameter attached on the PI tape, corresponding to the patterns in figure S1 a-d.**

**
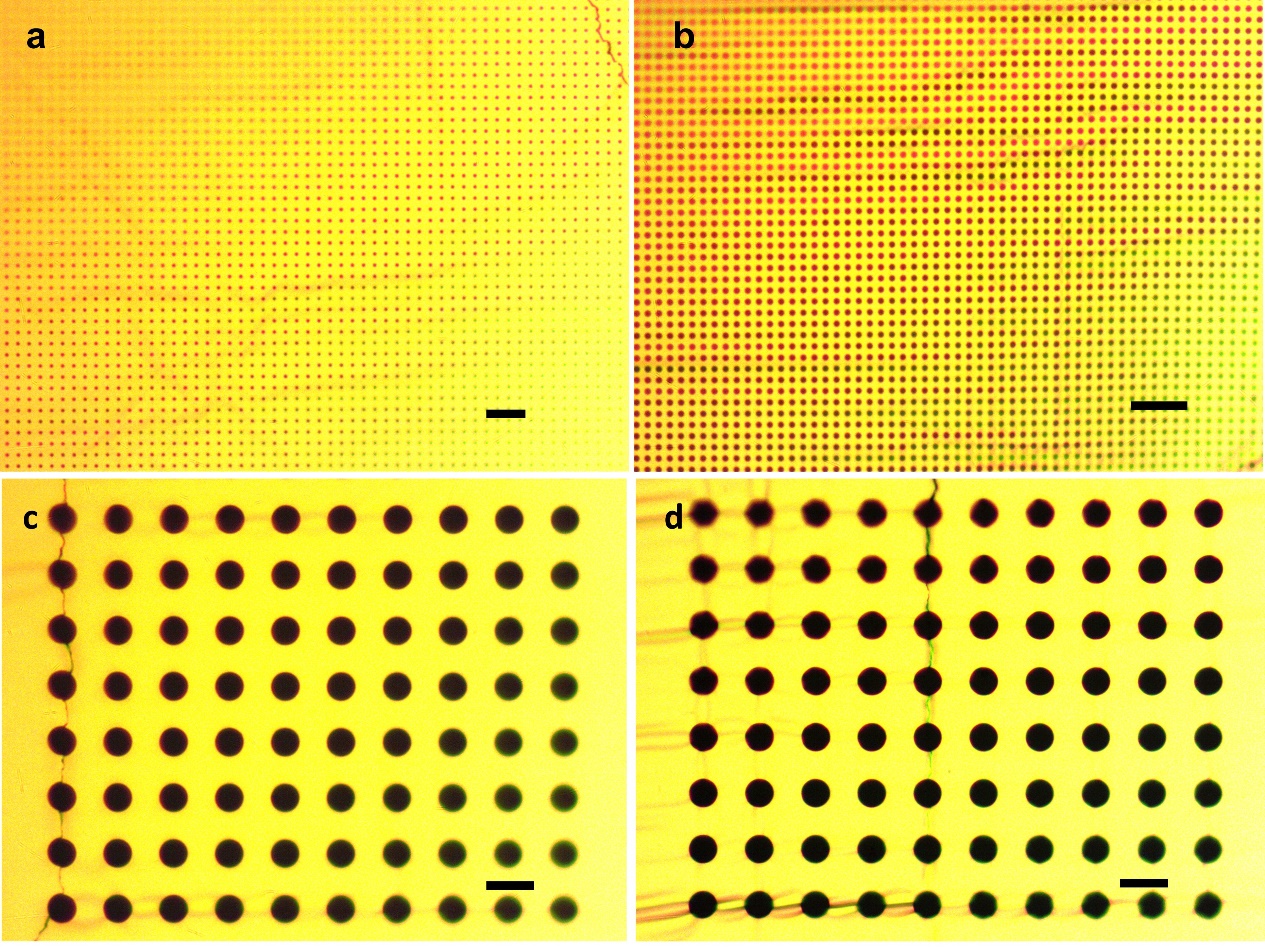
**

**Fig. S3 a**, 500 nm. **b**, 1 μm. **c**, 5 μm. **d**, 10 μm. Rugged topography on the surface of gold film reveals unevenness of adhesive tape. Meanwhile, cracks of gold film in the micrographs was attributed to malleability limitation of gold thin film. Scale bar: 5 μm (a), 10 μm (b and c), 20 μm (d).

**4. (I) Mixed-mode cohesive zone model**. Cohesive zone models were widely used in the area of fracture mechanics. In some of these models, the constitutive behavior of the interface was assumed as a bi-linear Traction-Separation ($T-\delta$) law composed of an elastic stage and a softening stage (Figure S4). The interface traction $T$ depends on the separation vector $\delta$, i.e., the relative displacement of two points that are initially located at the same position on the opposite surfaces of the interface. Before reaching the peak traction (correspondingly, the interface stress reaching strength $\bar{\sigma}$), the traction–separation relation is linear and reversible. Once the peak traction is reached, the traction decreases to zero in a softening manner as separation increases. The separation and traction vectors ($\delta$,$T$) can be mainly decomposed into ($\delta_{n}$,$T_{n}$) normal to the interface, the mode-I contribution, and ($\delta_{t}$,$T_{t}$) vertical to the crack front, the mode II contribution. In most cases, the mode-II interface fracture is more difficult to occur than the mode-I fracture. The constitutive law in Figure S4 is completely determined by the adhesion work $W_{ad}$ (the area of triangle) and interfacial strength $\bar{\sigma}$ (the height of triangle), considering the interface fracture behavior is insensitive to the detailed shape of the $T-\delta$ curve.^[1]^ In the problem studied herein, the interface strength is crucial, rather than adhesion work.

**
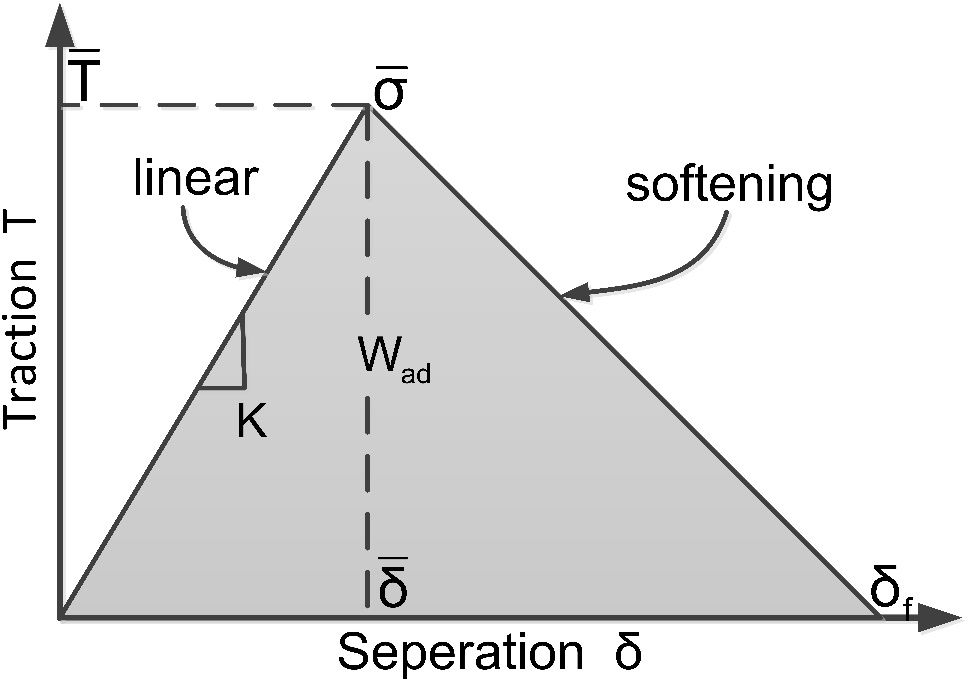
**

**Fig. S4** Traction-Separation (T-δ) law of cohesive zone model

**(II) Analysis of the failure process in the condition of zero slant angle and small feature sizes.** Figure 3d shows the simulation results of the Traction-Tensile Displacement curves, for a small feature size of 1 μm and no slant angle. At a relative small feature size, the stress states of interfaces were uniform and the failure tends to occur simultaneously at different locations of the interface. Thus, the failure thresholds can be calculated by multiplying the interface strengths and areas,

$\left\{\begin{matrix} \bar{T}_{Au/Tape,n}=\frac{1}{4}\pi D^{2}\bar{\sigma}_{Au/Tape,n} \\ \bar{T}_{Au/PMMA,n}=\frac{1}{4}\pi D^{2}\bar{\sigma}_{Au/PMMA,n} \\ \bar{T}_{Au/PMMA,t}=\pi Dw\bar{\sigma}_{Au/PMMA,\mathrm{t}} \end{matrix}\right.$, (Eq.S1)

where *D* is the diameter of the disk, $w$ is the covered height and $\bar{\sigma}$ is the corresponding interface strength. During the entire computational process, the equilibrium equation, $T_{Au/PMMA}\text{=}T_{Au/PMMA,n}+T_{Au/PMMA,t}=T_{Au/Tape,n}$, was always satisfied for the Au disk. For the Au/Tape interface, the tensile distance was exactly the separation of two opposite surfaces, and therefore, the shapes of Traction-Tensile Displacement curves for $T_{Au/PMMA}$ and $T_{Au/Tape,n}$ in Figure 3d are bi-linear, similar to that of the cohesive model. In region A of the diagram, the force between Au and PMMA was supported mainly by the top Au/PMMA interface. As a result, the normal traction $T_{Au/PMMA,n}$ increased linearly with the tensile displacement, and it no longer changed once it attained the threshold $\bar{T}_{Au/PMMA,n}$. In the region B, the tangential force $T_{Au/PMMA,t}$ grew in the same rate of $T_{Au/Tape,n}$ to support the force increment, until the separation reaches $\delta_{2}$, where the threshold $\bar{T}_{Au/Tape,n}$ was attained and the Au/Tape interface started to damage. Finally, in region C, the Au/Tape interface gradually debonded until the full delamination.

**(III) Analysis of the failure process in the condition of non-negligible slant angles and relatively large feature sizes**. Figure S5a shows the simulation results of Traction-Tensile Displacement curves with slant angles of 0 ^o^, 5.5 ^o^, 5.8 ^o^, 6 ^o^, with a fixed diameter of 10 μm, each of which represents the resultant traction on the Au/Tape (or Au/PMMA) interface. In region A of the diagram, the four curves were almost coincident, attaining a peak followed by a sharp decrease, which was due to the failure of a large area in the middle region of the disk. Then the curves rose slowly, during which the applied load was mainly supported by the sidewall. Next it had two possible cases - the tape is damaged after reaching its failure strength (the * point in Figure S5a) as shown by the green line; the gold disk was pulled out from PMMA and the normal contact force at the side surface of PMMA diminished rapidly. The cross-sectional views of FEA at four representative states marked in Figure S5a are shown in Figure S5b.

**(IV) Analysis of the dominant effect of the slant angle.** From the phase diagram in terms of the diameter and the slant angle in Figure 3e, it can be seen that the influence of slant angle will increase sharply when it attains ~5.5°. To illustrate the dominant effect of the slant angle from the viewpoint of force and strain energy, the maximum resistance force and the maximum strain energy during the pull-out process under different slant angles (0°, 4°, 5°, 5.5°, 5.8°, 5.9°) were obtained and shown in Figure S5c and d, respectively. Both the peak force & strain energy increase sharply after the slant angle attains ~5.5°, and become much more sensitive to the slant angle beyond this angle. It is noteworthy that the strain energy of gold disk increases much faster than that of PMMA as the slant angle reaches ~5.5°. This arises mainly from the slant side wall that possessed a strong constraint, especially at relative large slant angles, such that the pull-out process needs to overcome much more additional strain energy. The strong constraint beyond θ ≈ 5.5° can be attributed to the distinct difference of deformations. Figure S5e and f provides additional information of detailed deformations in the cases of θ = 4° and 6°. The PMMA undertakes main deformation for the demand of the pull-out for the gold disk under the small slant angle (θ = 4°), which can be well demonstrated by a contour of displacement U1 in the cross-sectional view in Figure S5e. In this situation, the compressional force on the near-vertical side wall will effectively shorten the radial dimension of PMMA because of the small elasticity modulus of PMMA resist and the negligible slant angle. When slant angle is larger than ~5.5°, the incline of side wall becomes non-negligible, and the PMMA will not occur large shortening in radial direction under compression on side wall (not more than gold disk), as shown in Figure S5f. In this situation, the PMMA and gold disk are limited by "self-locking", and the cost of the pull-out for the gold disk increases rapidly.


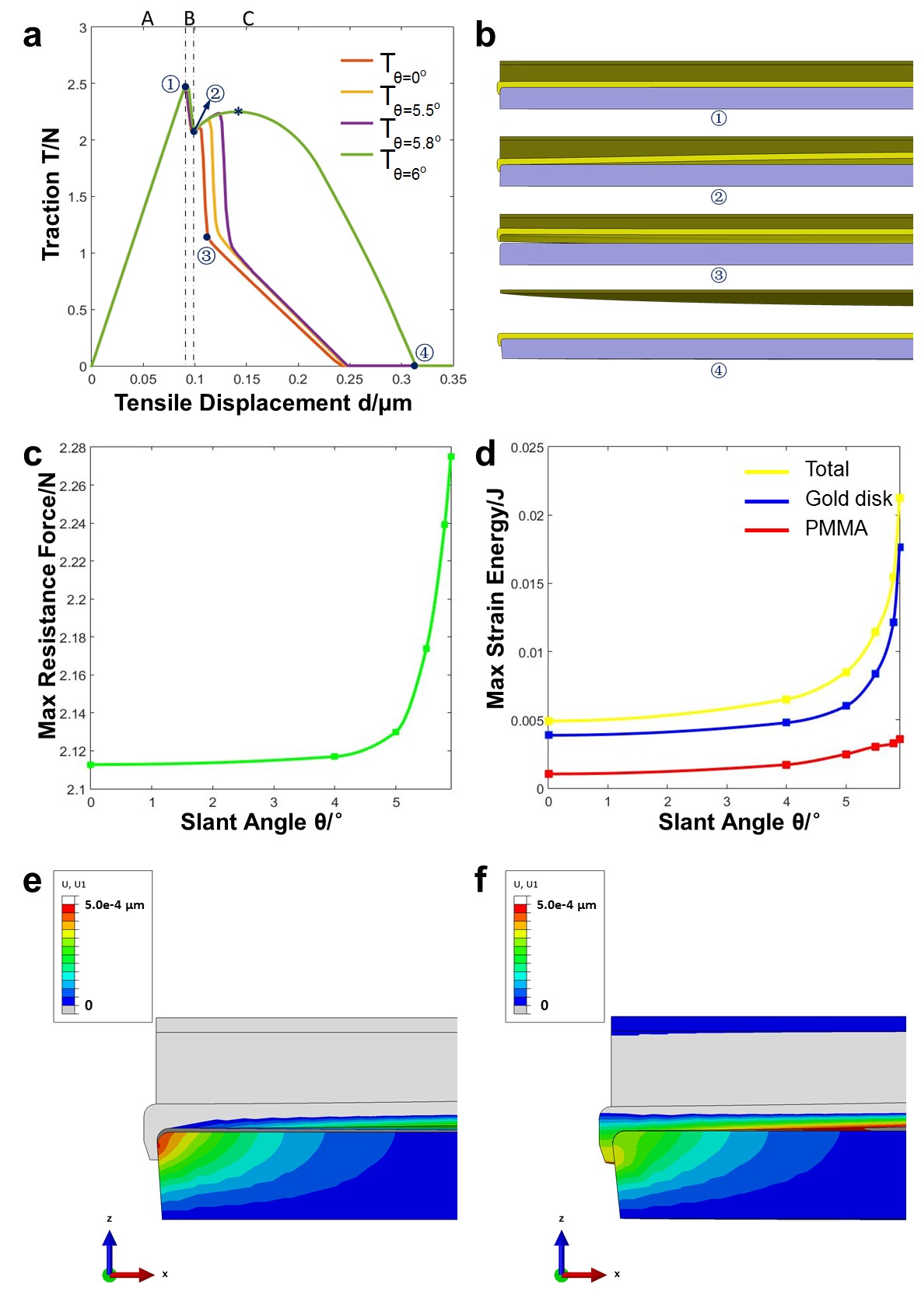


**Figure S5.** **Failure process on the condition of large feature size and slant angle. a**, Plot of calculated resultant tractions versus tensile displacement with four different slant angles during the entire peeling process. **b**, Cross-sectional views of FE model corresponding to four states marked out in **a**. **c**, Plot of calculated maximum resistance force versus slant angle and its fitting curve. **d**, Plot of calculated strain energy versus slant angle and fitting curves. **e**, Contour of displacement U_1_ in cross-sectional view under *θ* = 4° when the Au disk is going to be pulled out. **f**, Contour of displacement U_1_ in cross-sectional view under *θ* = 6° and the same load in **c**.

1. **Characterization and optical properties of the concealable miniature.**

**
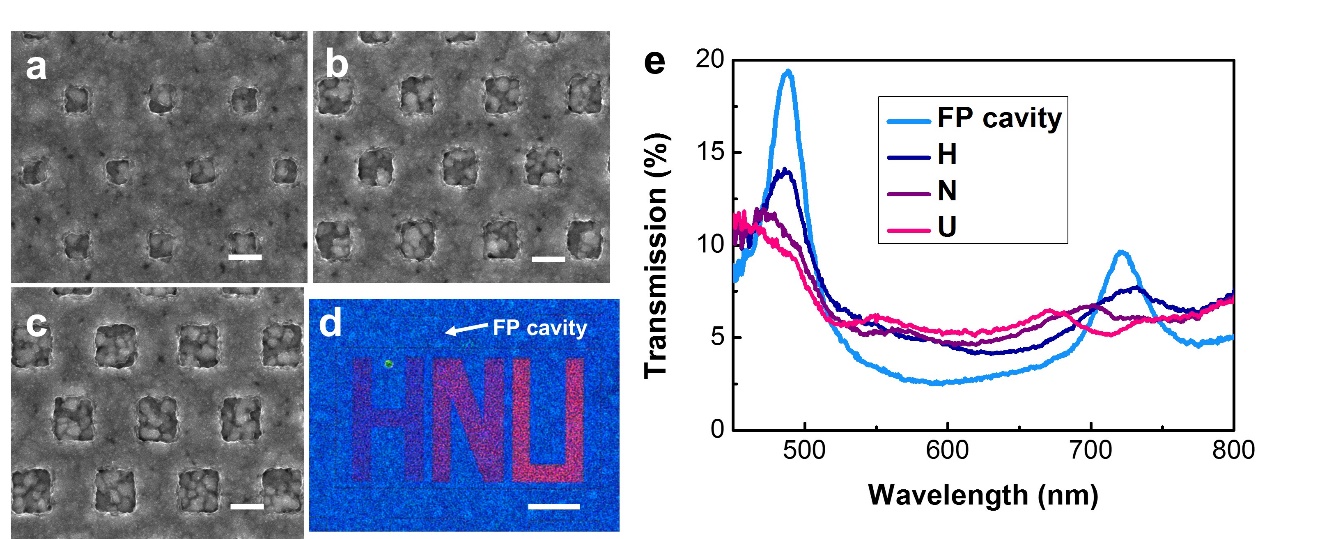
**

**Fig. S6 a-c,** SEM images of three characters H, N and U, respectively. **d,** Transmission optical micrograph of characters consisting of plasmonic nanoholes array before stripping. **e,** Transmission spectra of Ag-PMMA-Ag sandwich-type FP cavity and three characters consisting of triangular-lattice nanohole array. Scale bar: 200 nm (a-c), 20 μm (d).

1. **Optical properties of gold dimer for SERS application.**


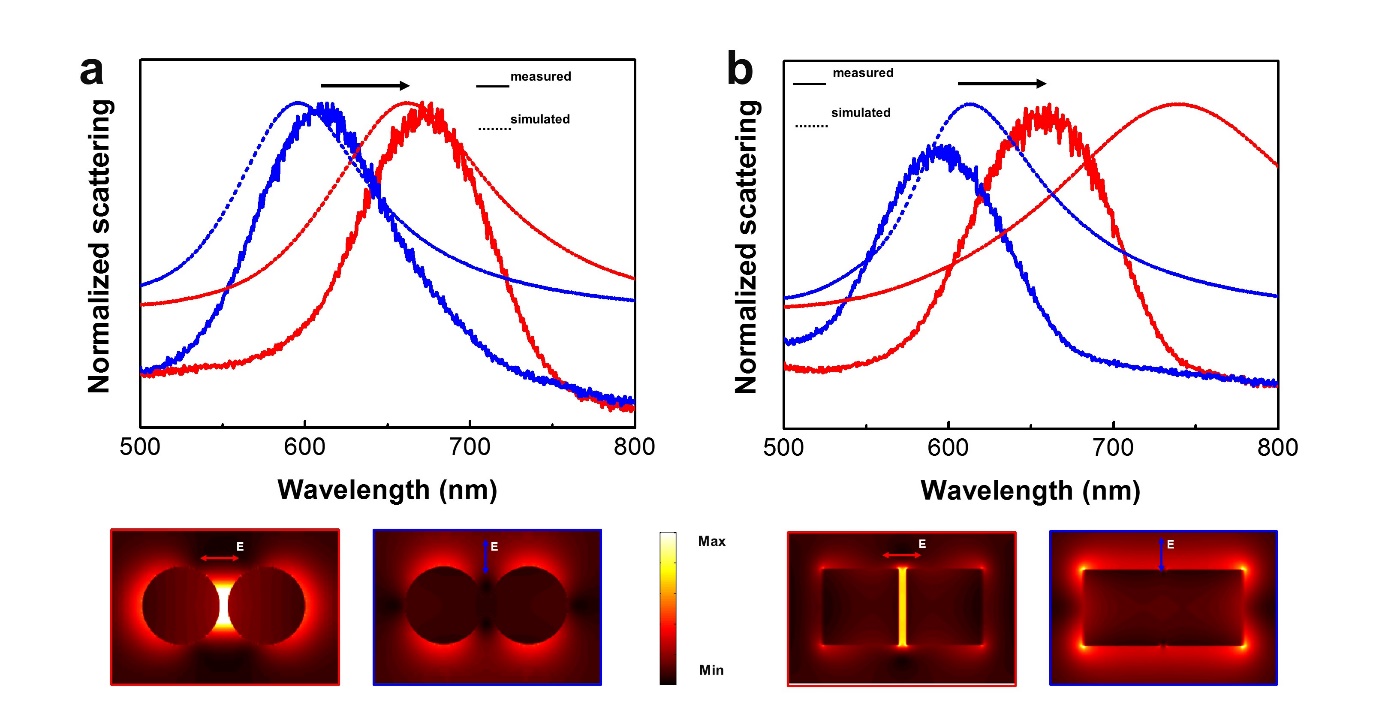


**Fig. S7 a-b,** Normalized scattering spectra of individual gold nanodisk dimer and nanosquare dimer with horizontal (red) and vertical (blue) polarized collection, respectively. Experimental results (solid line) agrees well with calculated ones (dot line). The black arrows highlight the red-shift of surface plasmon resonance with presence of tiny gap. Simulated electromagnetic field profiles under two different excited polarized directions were shown at the bottom.

1. **Near-field optical properties of nanodisk dimer with tiny gap.**

**
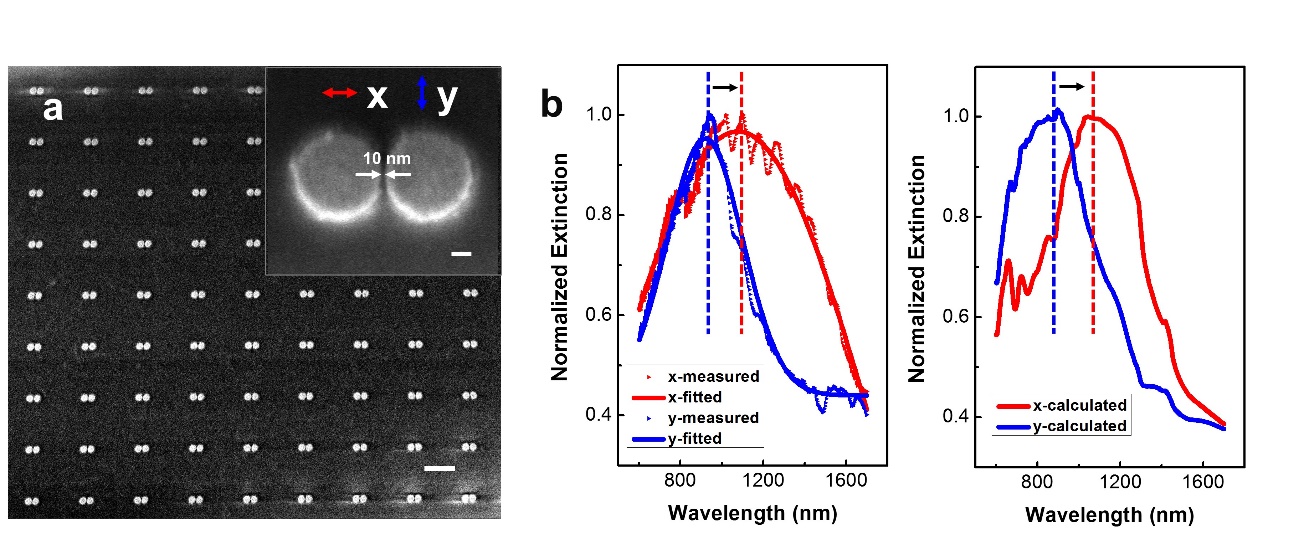
**

**Fig. S8 a,** SEM image of an array consists of 250-nm-diameter gold nanodisk dimer with 10 nm gap on quartz substrate. **b,** Measured (left) and calculated (right) normalized extinction spectra of disk dimer array in near-infrared region with horizontal (red) and vertical (blue) polarized collection. Scale bar: 1 μm (a), 50 nm (inset in a).

**Reference**

1. Hui CY, Ruina A, Long R, Jagota A, *J. Adhes.* **87**, 1–52 (2011).
